# Severe Hypokalemia Associated With Piperacillin–Tazobactam Use

**DOI:** 10.1155/crin/1957735

**Published:** 2026-09-03

**Authors:** Angelina Kravcik, Jason Zelt, Stephen Kravcik

**Affiliations:** ^1^ Faculty of Health Sciences, University of Ottawa, Ottawa, Ontario, Canada, uottawa.ca; ^2^ Department of Medicine, University of Ottawa, Ottawa, Ontario, Canada, uottawa.ca

## Abstract

Piperacillin–tazobactam is a generally well‐tolerated broad‐spectrum antibacterial. Mild hypokalemia may occur with piperacillin–tazobactam therapy as a result of increased renal potassium losses. Severe hypokalemia is rare. We report here a case of very severe hypokalemia during piperacillin–tazobactam therapy, refractory to aggressive potassium replacement.

## 1. Introduction

Piperacillin is a semisynthetic, broad‐spectrum, ampicillin‐derived ureidopenicillin antibiotic with excellent activity against Gram‐negative organisms, including *Pseudomonas aeruginosa* but is susceptible to *β*‐lactamase degradation. The addition of tazobactam, a *β*‐lactamase inhibitor, restores activity against many *β*‐lactamase‐producing Enterobacterales and extends its coverage to include methicillin‐susceptible *Staphylococcus aureus*.

Piperacillin–tazobactam is generally well tolerated and is widely used for the treatment of severe infections, including intraabdominal infections, hospital‐acquired pneumonia, and as empiric treatment for sepsis. Both piperacillin and tazobactam are primarily renally excreted via glomerular filtration. As a nonresorbed anion, piperacillin has the potential to promote renal potassium wasting and in rare cases, clinically significant hypokalemia. We present here a case of severe hypokalemia occurring with piperacillin–tazobactam use and refractory to exogenous potassium supplementation. Formal written consent was obtained from the patient’s next of kin to report this case.

## 2. Case

The patient was an 81‐year‐old male with a history of Waldenstrom’s macroglobulinemia, chronic hepatitis B, dyslipidemia, benign prostate hypertrophy, and a nonfunctioning pituitary adenoma. His medications included hydrocortisone 20 mg in the morning, 10 mg in the evening, l‐thyroxine 0.1 mg daily, testosterone 2.5 g topically daily, pantoprazole 40 mg daily, tenofovir disoproxil 300 mg daily, and tamsulosin 0.4 mg daily. He also received maintenance rituximab. One week prior to admission, serum potassium was 3.7 mmol/L and creatinine was 60 μmol/L (creatinine clearance 76 mL/min).

He was admitted to hospital with fever and progressive dyspnea over several days. A chest X‐ray was performed which showed bilateral airspace opacity in the left mid and right lower zones. A respiratory viral panel returned positive for COVID‐19, and due to mild hypoxemia requiring supplemental O₂, was started on dexamethasone 6 mg daily. He was also treated empirically for a potential superimposed bacterial pneumonia with piperacillin–tazobactam 3.375 g IV every six hours and vancomycin 1 g every 12 hours (vancomycin was discontinued after four doses).

**FIGURE 1:**
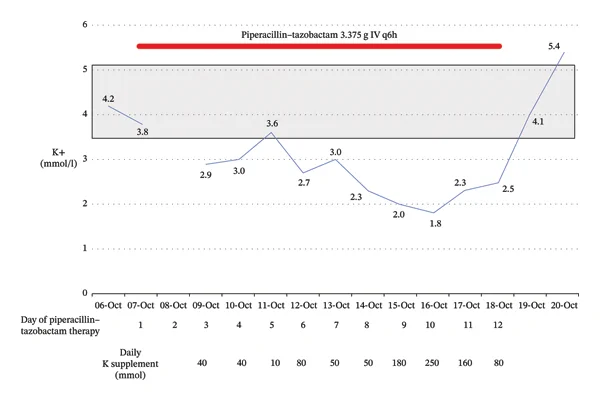
Timeline of serum potassium level. Serum potassium concentration (mmol/L). Piperacillin–tazobactam dosing indicated at top of graph; daily potassium supplementation noted at bottom. Gray box denotes normal serum potassium (3.5–5.1 mmol/L).

Two days later, worsening hypokalemia developed, with a nadir potassium of 1.8 mmol/L on Day 10 of therapy (Table 1, Figure 1). Potassium levels remained extremely low despite aggressive oral and intravenous potassium replacement of up to 250 mmol/day. In this period, his serum magnesium ranged between 0.60 and 0.79 mmol/L (normal 0.66–1.07 mmol/L; oral magnesium hydroxide 252 mg given, three doses total) and the creatinine remained normal (62–79 μmol/L, normal 62–100 μmol/L). Metabolic alkalosis developed (Table 1). On Day 9 of piperacillin–tazobactam therapy, random urinary potassium:creatinine ratio was elevated (15.7 mmol/mmol, normal 5.2–10.6 mmol/mmol) with simultaneous serum potassium 2.0 mmol/L; with this, urinary potassium was 55 mmol/L and urinary sodium 74 mmol/L. Venous blood gas measured the same day demonstrated pH 7.42, pCO_2_ 47 mm Hg and HCO_3_ 30 mmol/L, with serum HCO_3_ 32 mmol/L.

**TABLE 1:** Timeline of biochemical changes, piperacillin–tazobactam, and corticosteroid use.

Date	Serum K (mmol/L)	Serum HCO_3_ (mmol/L)	Serum Mg (mmol/L)	Piperacillin–tazobactam use	Steroid dose
Oct 6	4.2	23	0.79		HC 100 mg IV × 1, HC 30 po mg/d
Oct 7	3.8	25	0.76	+	Dex 6 mg/d (oral)
Oct 8				+	Dex 6 mg/d (oral)
Oct 9	2.9	29	0.77	+	Dex 6 mg/d (oral)
Oct 10	3.0	29		+	Dex 6 mg/d (oral)
Oct 11	3.6	29		+	Dex 6 mg/d (oral)
Oct 12	2.7	26	0.74	+	Dex 6 mg/d (oral)
Oct 13	3.0	28		+	Dex 6 mg/d (oral)
Oct 14	2.3	29	0.66	+	Dex 3 mg/d (oral)
Oct 15	2.0	32	0.60	+	Dex 3 mg/d (oral)
Oct 16	1.8	34	0.71	+	Dex 3 mg/d (oral)
Oct 17	2.3	37	0.69	+	Dex 3 mg/d (oral)
Oct 18	2.5	36	0.64	+	Dex 3 mg/d (oral)
Oct 19	4.1	33	0.75		Dex 20 mg/d (IV)
Oct 20	5.4	30	0.81		Dex 20 mg/d (IV)
Oct 21	3.9	29			Dex 20 mg/d (IV)
Oct 22	4.0	34			Dex 20 mg/d (IV)
Oct 23	3.6	32			Dex 20 mg/d (IV)

*Note:* Piperacillin–tazobactam 3.375 g IV q6h. HC = hydrocortisone. Dex = dexamethasone.

Clinically, he was very dyspneic and generally unwell during this period. His EKGs demonstrated new PR and QTc prolongation compared to baseline, with episodes of atrial bigeminy and sinus pauses.

Other risk factors for acute hypokalemia were reviewed. He did not receive other commonly offending agents including insulin, beta‐adrenergic agonists, laxatives, diuretics, or aminoglycosides. Chronic tenofovir dosing was continued; urinary losses of low molecular weight proteins were not quantified; he was not glycosuric, and serum phosphate levels were between 0.73 and 0.87 mmol/L in this period (normal 0.78 – 1.38 mmol/L). TSH and blood sugar measurements were normal.

With the substitution of meropenem for piperacillin–tazobactam, the potassium level rapidly returned to normal or slightly above (Figure 1), with resolution of his EKG changes. The patient continued to clinically worsen from presumed organizing pneumonia; increased dosing of dexamethasone 20 mg once daily and then twice daily for worsening suspected organizing pneumonia did not significantly drop serum potassium levels. The patient passed away in hospital from progressive respiratory failure about 1 month after the episode of severe hypokalemia.

## 3. Discussion

This case illustrates the rapid development of severe, potentially life‐threatening hypokalemia during treatment with piperacillin–tazobactam for potential community‐acquired pneumonia occurring with COVID‐19 infection. The dose of piperacillin–tazobactam (3.375 g IV every six hours) was appropriate for the CrCl (76 mL/min). Renal potassium loss in the presence of severe hypokalemia was documented. Hypokalemia resolved when the piperacillin–tazobactam was discontinued and meropenem (not associated with hypokalemia) started.

Alternative causes of hypokalemia were systematically excluded. There was no evidence of reduced oral intake, or intracellular redistribution of potassium (beta sympathetic drive, insulin exposure, or refeeding syndrome), and gastrointestinal losses were not clinically apparent. Solute diuresis and diuretic use were not present in this case.

Admittedly, concomitant corticosteroid use and mild metabolic alkalosis may have contributed to the development of hypokalemia but were likely minor contributors as higher doses of dexamethasone and recurrent metabolic alkalosis later in the hospital course did not significantly decrease serum potassium levels, and the period of severe hypokalemia occurred only during piperacillin–tazobactam therapy, resolving with this medication’s discontinuation. Mild hypomagnesemia (possibly related to pantoprazole use, continued through this hospitalization) may have contributed to the development of hypokalemia, as it impairs renal potassium conservation and the response to potassium replacement. Finally, chronic tenofovir use may have predisposed to renal potassium and magnesium wasting through the development of a proximal tubulopathy. Unfortunately, renal losses of low molecular weight proteins or phosphate were not quantified, but there was no glycosuria, and serum phosphate levels were normal or only slightly reduced. The hypokalemia resolved with piperacillin–tazobactam discontinuation, with ongoing tenofovir use. Application of the details of this case to the Naranjo Adverse Drug Reaction Probability Scale [1] yields a score of 7, indicating the presence of a probable adverse drug reaction (Table 2).

**TABLE 2:** The Naranjo ADR Probability Scale.

Questions	Yes	No	Don’t know	Score
1. Are there previous conclusive reports on this reaction	+1	0	0	+1
2. Did the ADR appear after the suspected drug was administered?	+2	−1	0	+2
3. Did the ADR improve when the drug was discontinued?	+1	0	0	+1
4. Did the ADR appear with rechallenge?	+2	−1	0	0
5. Are there alternative causes for the ADR?	−1	+2	0	+2
6. Did the reaction appear when placebo was given?	−1	+1	0	0
7. Was the drug detected in blood at toxic levels?	+1	0	0	0
8. Was the reaction more severe when the dose was increased, or less severe when the dose was decreased?	+1	0	0	0
9. Did the patient have a similar reaction to the same or similar drug in any previous exposure?	+1	0	0	0
10. Was the ADR confirmed by any objective evidence?	+1	0	0	+1
Total score				7

*Note:* Interpretation of scores, ≥ 9 Definite (the reaction (1) followed a reasonable temporal sequence after drug exposure had been established and body fluids or tissues, (2) followed a recognized response to the suspected drug, (3) was confirmed by improvement on withdrawing the drug, and (4) reappeared on reexposure), 5–8 Probable (the reaction (1) followed a reasonable temporal sequence after a drug exposure, (2) followed a recognized response to the suspected drug, (3) was confirmed by withdrawal but not by exposure to the drug, and (4) could not be reasonably explained by the known characteristics of the patient’s clinical state), 1–4 Possible (the reaction (1) followed a temple sequence after a drug disclosure, (2) possibly followed a recognized pattern to the suspected drug, and (3) could be explained by characteristics of the patient’s disease), 0 Doubtful (the reaction was likely related to factors other than a drug).

Piperacillin, like penicillin, is a nonresorbed anion that may lead to kaliuresis. Piperacillin reaches the distal nephron as a large, poorly resorbable anion; because it cannot follow Na+ resorption it increases lumen electronegativity in the aldosterone‐sensitive distal nephron/collecting duct, augmenting the driving force for *K*+ and *H*+ secretion, hence both kaliuresis and a tendency to metabolic alkalosis [2].

The association between piperacillin‐tazobactam use and hypokalemia is increasingly recognized, although severe cases remain uncommon. Indeed, published data suggests that hypokalemia is not rare: one study demonstrated a decline of potassium to ≤ 3 mmol/L in 30/168 patients, with risk factors included lower baseline potassium levels and diarrhea [3]. Another demonstrated that hypokalemia occurred in 24.8%, and was associated with increasing age [4]. A larger retrospective cohort study found that older age, female sex, longer duration of therapy and higher daily dose, were all important risk factors for hypokalemia during treatment with piperacillin–tazobactam [5].

Despite this, reports of profound hypokalemia are limited to few case reports [6, 7]. It is currently unknown why some patients develop severe or resistant hypokalemia while treated with piperacillin–tazobactam; in our case, other factors (mild hypomagnesemia and tenofovir use) may have contributed. Treatment in such cases would sensibly include potassium supplementation and piperacillin–tazobactam discontinuation. The benefit of treatment with amiloride or other potassium‐sparing diuretic, in patients who require continuation of the antibiotic, is uncertain and appears unexplored in the medical literature.

In summary, the literature suggests that it is common to have serum potassium levels decline with piperacillin–tazobactam use. Very severe hypokalemia may occur, as in our case, but is rare. Routine electrolyte monitoring when treating with piperacillin–tazobactam would seem prudent, particularly in patients with additional risk factors or during prolonged courses of therapy.

## Funding

No funding was received for this manuscript.

## Conflicts of Interest

The authors declare no conflicts of interest.

## Data Availability

The data that support the findings of this study are available on request from the corresponding author. The data are not publicly available due to privacy or ethical restrictions.
